# Mediterranean Diet Adherence and Eating Disorders in Spanish Nurses with Shift Patterns: A Cross-Sectional Study

**DOI:** 10.3390/medicina57060576

**Published:** 2021-06-04

**Authors:** Belén Leyva-Vela, Cristina Reche-García, Juan José Hernández-Morante, María Martínez-Olcina, Laura Miralles-Amorós, Alejandro Martínez-Rodríguez

**Affiliations:** 1 Department of Health, Vinalopó University Hospital, 03293 Elche, Spain; bmleyva@vinaloposalud.com; 2 Faculty of Nursing, Catholic University of Murcia, 30107 Murcia, Spain; creche@ucam.edu (C.R.-G.); jjhernandez@ucam.edu (J.J.H.-M.); 3 Faculty of Health Sciences, University of Alicante, 03690 Alicante, Spain; maria.martinezolcina@ua.es (M.M.-O.); lma52@alu.ua.es (L.M.-A.); 4 Department of Analytical Chemistry, Nutrition and Food Science, Faculty of Sciences, University of Alicante, 03690 Alicante, Spain; 5 Alicante Institute for Health and Biomedical Research (ISABIAL Foundation), 03010 Alicante, Spain

**Keywords:** mental health, eating disorders, nursing, shift work schedule, nutrition, Mediterranean diet, healthy diet, dietary risk

## Abstract

*Background and Objectives:* Shift work has a significant influence on the mental health of workers. Nursing is characterised by a rotational work system. This study aimed to determine whether there was a link between adherence to the Mediterranean diet (MD) and the risk of suffering an eating disorder (ED) in nurses according to their work shift. *Materials and Methods:* A total of 240 women (nurses and nursing assistants) were evaluated and completed the PREDIMED-PLUS questionnaire on adherence to the MD and the EAT-26 (Eating Attitude Test, 26 items). *Results:* The results indicate that there are no differences in adherence to the MD depending on the work shift, being that adherence to the diet is already low. Statistically significant differences appear depending on the work shift in the following dimensions: restrictive behaviours and bulimia subscales (presenting higher scores in the 7-h rotating shift versus the fixed morning shift or 12-h rotating shift) and for total EAT-26 score. *Conclusion:* Whilst they do not condition adherence to a MD, the nursing shifts that are the most changing in terms of time patterns may condition restrictive behaviours and compensatory risk behaviours related to an ED.

## 1. Introduction

Today’s society presents health demands derived from specific circumstances: demographic and economic factors, prevalent pathologies, information technologies, population movements, health habits and citizens’ rights, among others. The nursing profession, through quality care, ensures a professional practice based on the professional values legitimised in the eyes of society, responding to the health demands derived from the aforementioned factors [1,2].

In order to provide a continuous service to satisfy these needs, it is necessary to create different work shifts in hospitals. According to the workers’ statute, shift work is classified into: discontinuous (interruption of work on a regular basis at night and on weekends), semi-continuous (24 h a day are covered, resting on Sundays) and continuous systems (work is performed uninterruptedly every day of the week) [2].

The performance of the nursing profession, by its nature, has implications in terms of health and safety at work that require physical and mental effort in biologically contradictory schedules, such as the establishment of the rotational work system, which has become a traditional activity, a frequent practice and, therefore, a particular and formally established characteristic of this profession. This has a direct impact on the daily lives of healthcare workers, posing risks to health and quality of life [3].

Furthermore, it modifies activities such as leisure time, family, and social relationships [4,5]. Therefore, nursing professionals must take these conditions into account and try to organize themselves both physically, psychologically, and socially [6]. In the first aspect, the deterioration of physical health by altered eating habits and sleep disorders is observed. Moreover, in the long term, it is caused by cardiovascular and neuropsychic alterations [7,8].

Shift work exposes workers to these cardiovascular alterations due to irregular sleep patterns, which cause fatigue and negatively affect physiological functions [9]. During and after night shifts, changes in heart rate, elevation of blood pressure and arrhythmias can also be observed due to altered catecholamine excretion and elevations in serum cholesterol, uric acid and potassium [4].

On the other hand, alterations in eating habits have been observed, in terms of the rhythm of eating, as well as the quality and quantity, increasing the consumption of soft drinks, snacks, sandwiches, caffeine and sweets, among others, in addition to the consumption of stimulants, such as caffeine or nicotine, to combat sleep [10,11].

The schedule of food intake is modified, favouring fast, cold meals and even not eating [6]. Gastrointestinal problems are frequent [4] due to the low consumption of a diet rich in fibre and a high consumption of lipids [8]. Flatulence, heartburn, digestive ulcers, constipation, haemorrhoidal problems and diarrhoea are usually present [4].

In various investigations [10,12,13], it has been concluded that working at night is a risk factor for the health and quality of life of healthcare workers. However, the nutritional status and eating habits of hospital workers, as in the case of nursing staff, should also be considered. It has been observed that the distribution of energy and nutrient intake changes depending on the work shift [14], with an association between lipid and protein intake for workers belonging to the rotating shift [13,15], highlighting the consumption of beef, eggs, juices and pasta [16].

This does not correspond to healthy eating patterns such as the Mediterranean diet (MD). The MD, i.e., a diet rich in vegetables, fruits, greens, nuts and cereals, with low or moderate consumption of dairy products (yogurt, cheese) and poultry meat (mainly chicken) and limited consumption of red meat, sweets and bakery products, is one of the healthiest dietary patterns to follow, since it has been observed that health habits and lifestyle habits are intimately linked [13,17]. Higher adherence to the MD is associated with a lower risk of mortality, cardiovascular disease, cancer incidence, Parkinson’s disease and Alzheimer’s disease [17,18,19]. Adherence to the MD has been shown to be moderately low, even in Mediterranean countries. Sedentary behaviours with unhealthy dietary choices aggravate the harmful effects on health, as demonstrated in women who are mainly involved in health care and education [20]. The existence of alternating work shifts promotes the occurrence of these behaviours and their negative consequences [17,18].

These negative consequences include psychological consequences. In a recent systematic review conducted by Devonport et al. [21], it has been shown that negative emotions can cause feelings of satiety, leading to a decrease in the amount of food consumed. Conversely, the amount consumed may contribute to alleviating the distress of negative emotions. This phenomenon has been explained as “emotional eating”. Emotional eating facilitates uncontrolled eating and is caused by an absence of adaptive emotional regulation. This is associated with a deficit in impulse control [22], which can lead to the development of severe alterations in eating behaviour, resulting in eating disorders (ED) [23,24].

Therefore, eating behaviour can be altered by shift work, especially when night work is involved. The number of cases of EDs has doubled in recent years, reaching a worldwide prevalence of 7.8% in 2018 [25]. According to continents, the prevalence were 4.6% (2.0–13.5%) in America, 2.2% (0.2–13.1%) in Europe, and 3.5% (0.6–7.8%) in Asia [25].

The short-term effects are related to a lack of energy supply, while the long-term effects refer to a lack of supply of essential nutrients, i.e., vitamins, minerals and essential amino acids and fatty acids that are necessary for the functioning of the senses and biochemical processes [26].

To date, there is little evidence regarding the relationship between ED and the MD in health professionals. Only the SUN cohort studied these aspects in women, finding inverse relationships between adherence to the MD and ED. However, no differences in adherence were found between participants with and without ED, and further research is needed [27].

The aim of this study is to evaluate the effects of different work shifts of nursing staff on adherence to the MD and EDs, with the following specific objectives: a) to describe the adherence to the MD of nursing staff as a function of different work shifts; b) to know the risk of EDs in nursing staff as a function of their work shift. The main hypothesis of this work is that rotating shifts may have a negative influence on adequate eating habits, as well as affecting a greater predisposition to present an ED.

## 2. Materials and Methods

### 2.1. Study Design

This was a cross-sectional observational study with chain sampling, where contact was established with the supervision of different sections of different health departments in the province of Alicante, Spain. The study was carried out in accordance with international recommendations on clinical research and the ethical code of the World Health Organization (Declaration of Helsinki). All participants signed the informed consent form, which was approved by the Ethics Committee of the Catholic University of Murcia (Code CE022008).

### 2.2. Participants

A total of 240 female nursing staff (nurses and nursing assistants) (37 ± 10 years) from health departments in the province of Alicante participated in this study. Women who were pregnant or under medical or psychiatric treatment were excluded. The study population was divided according to the work shift they had performed during the last year, which could be: (a) fixed morning shift (FMS) (Monday to Friday from 8 am to 3 pm); (b) 7 h rotating shift (RS7) (2 days from 8 am to 3 pm, 2 days from 3 pm to 10 pm, 2 days from 10 pm to 8 am and 3 days off); (c) 12 h rotating shift (RS12) (1 day from 8 am to 8 pm, 1 day from 8 pm to 8 am and 3 days off).

### 2.3. Data Collection

#### 2.3.1. Sociodemographic and Mediterranean Diet Adherence

An ad hoc questionnaire was prepared with the aim of determining different data, integrating questions related to age, sex, height, weight, work shift (FMS, RS7, RS12), pregnancy, and medical or psychiatric treatment.

Adherence to Mediterranean dietary patterns was assessed by a modified version of the previously validated questionnaire used in the PREDIMED trial. Registered dietitians administered the PREDIMED-PLUS (17-item MedDiet) questionnaire measuring adherence to an energy-restricted MedDiet [28,29]. This questionnaire includes information on the characteristics of the MD and the consumption of: (1) oil; (2) fruits; (3) vegetables; (4) white bread; (5) whole grains; (6) meat; (7) fats; (8) sugary drinks; (9) legumes; (10) fish; (11) pastries; (12) nuts; (13) white meats; (14) fried foods; (15) sweeteners; (16) refined cereals; (17) wine. Compliance with food habits scored 1 for every item, but otherwise scored 0. Therefore, results of the 17-item MedDiet questionnaire ranged between 0 and 17. Terciles were made to define low, moderate or high adherence, ranging from 0 to 7, 8 to 10, and 11 to 17, respectively.

#### 2.3.2. Eating Disorders

The Eating Attitudes Test, in its reduced 26-item version (EAT−26) [30], which has been validated in Spanish women [31], was used for the evaluation as a possible screening for the future presence of an ED. Through the global computation of its 26 items, which are classified into three differentiated scales: (a) food restriction (avoidance of fattening foods and preoccupation with thinness); (b) bulimia and preoccupation with food; and (c) oral control [32], this self-administered questionnaire provides information on the possible risks associated with an ED. Among its items are questions that allow the detection of actions related to purging, diuretic use and binge eating, among others. The score to determine the test as positive, and alert the presence of a possible ED, either AN or BN, is 20 points out of a possible 78 [33]. The answers to this questionnaire are presented in the Likert format, from 1 (never) to 6 (always), whose order will represent a score of “0, 0, 0, 0, 1, 2 and 3 points” in all questions, except for items 1 and 25, “I like to eat with other people” and “I enjoy trying new and tasty foods”, in which the score is established in the reverse order. For reliability and internal consistency analysis, Cronbach’s Alpha (α) was performed for each of the subscales: dieting (α = 0.794); bulimia and food preoccupation (α = 0.752); and oral control (α = 0.732) for the total EAT-26 score (α = 0.825).

### 2.4. Statistical Analysis

For statistical analysis of the data, descriptive statistics (mean ± standard deviation), normality tests (Kolmogorov–Smirnov) and comparison of means were performed.

Comparison of means was performed by analysis of covariance (ANCOVA), establishing BMI as a co-variate. Bonferroni was established as a post hoc test. A significance level was established when the p-value was less than 0.05. The effect size was calculated using the partial eta-squared statistic (η^2^*p*), where the following indicators were established: small (0.01), medium (0.06) and large (0.14) [34]. The statistical analysis was carried out using the free statistical software JAMOVI (version 0.9).

## 3. Results

A total of 240 female nursing staff (nurses and nursing assistants) (37 ± 10 years), from health departments in the province of Alicante, Spain, participated. The average weight was between 62.8 kg and 63.5 kg, the average height was between 164.3 cm and 165.2 cm, and the average BMI was between 23–23.9 kg/m^2^. Table 1 represents the characteristics of the study sample.

Table 1 also shows the score obtained in the different groups following analysis of the data derived from the evaluation with the PREDIMED-PLUS questionnaire that assesses adherence to the MD. No significant differences were observed between groups (F = 0.065; *p* = 0.799; η^2^*p* = 0.001) and the effect size was insignificant. All groups showed an average score between 7–8 points. Table 2 shows descriptive statistics (frequencies and percentages) of the PREDIMED PLUS questionnaire items according to each group and its comparison. There are significant differences between groups in the consumption of white bread (item 4), with higher consumption in those nurses who have rotating shifts. There are also significant differences in the consumption of sugary drinks or sweetened fruit juices (Item 8), being lower in the RS12 group compared to RS7 and TMF. Finally, nurses with rotating shifts have a higher consumption of sweeteners instead of sugar (item 15).

Table 3 presents the comparison between the different subscales and total score obtained in the EAT-26 questionnaire for each of the population groups, according to work shift. In the restriction subscale, the RS7 group showed a significantly higher score than the FMS group (*p* ≤ 0.001) and the RS12 group (*p* = 0.020), with a medium effect size. Although the RS12 group also presented higher values than FMS, the difference was not significant. Regarding the bulimia subscale, similarly, the RS7 group presented significantly higher values compared to the FMS group (*p* ≤ 0.001) and the RS12 group (*p* = 0.05), also with a small effect size. In this case, the increased score of the RS12 group was not significantly higher than that obtained by the FMS group. In the oral control subscale, no significant differences were found between the different groups. Finally, in the total score of the EAT-26 questionnaire, the RS7 group again reported, although with a small effect size, a significantly higher score than the FMS group (*p* ≤ 0.001) and the RS12 group (*p* = 0.010), which did not present differences between them.

## 4. Discussion

The aim of the study was to describe adherence to the MD in nurses according to their different works shifts and the possible future presence of an ED in nurses depending on their work shift. The evaluated adherence to the MD in the nursing staff is low: the results are below the mean, without differences according to the work shift in which they performed.

On the other hand, it has been shown that women in the RS7 group show increased values compared to the FMS and RS12 groups in restriction, bulimia and total score on the EAT-26 questionnaire. Meanwhile, although the RS12 group also presents higher values than the FMS group, this is not sufficient to show a statistically significant difference between the groups. Thus, the nurses and nursing assistants evaluated through the self-reported questionnaire who have a RS7 work pattern present a greater risk of suffering from an eating behaviour disorder compared to the nursing staff on a FMS or RS12 work pattern, showing a greater avoidance of fattening foods, concern about thinness, bulimia symptomatology and concern about the foods they consume.

This could be due to the fact that more unstable shifts in terms of time patterns may condition their eating habits, and may consequently present incorrect behaviours, potentially predisposing to an ED, although there is no conditioning of the overall food selection, because no differences have been observed on the PREDIMED-PLUS questionnaire scores.

Souza et al. [35] also studied eating habits in jobs subjected to rotating shifts and fixed-shift work. They concluded that shift workers show changes in meal patterns, skipping more meals and consuming more food at unconventional times compared to regular shift workers.

Furthermore, both in a study conducted with nurses in USA [12] and in Japan [36], it was found that the distribution of food intake and food choice changed depending on the work shift, with a higher consumption of protein and fatty foods being observed in workers who belonged to the rotating group, highlighting the consumption of foods such as beef, eggs, juices and pasta, which suggests that the adherence to the MD is poor. Roskoden et al. [37] also conducted research on shift workers, studying their dietary habits, and established that shift workers tended to decrease the fibre content of their diet but increase the sucrose content. These findings coincide with the present investigation, since it has been observed that nurses who have rotating shifts have a higher consumption of bread and sugary beverages compared to those who work from 8 am to 3 pm.

Finally, a study by Gifkins et al. [26] on shift-working nurses found that there was an increase in food cravings, caffeine consumption and snacking behaviours during night shifts, besides an inability to consume enough fluids at work. Experienced nurses described more skipped meals at work [26]. These results do not concur with our research because the present study found no significant differences in eating habits according to work shift. However, it does correspond with the low quality of eating patterns in nursing staff.

Nevertheless, the results of the present research coincide with other studies, such as that carried out with nurses in Mérida [13], in which no significant differences were observed between those with rotating or fixed shifts in terms of eating habits. This could be explained by the difficulty of access to certain foods due to commitment to the patient and work overload.

Other researchers [38,39] also suggest that working shift schedules alters not only the usual meal schedule, but also the accessibility of healthy meals, which can generate multiple health-related problems.

On the other hand, previous research using the EAT-26 questionnaire in nursing staff showed similar values [2], although no significant differences were found in either the total score or the different subscales [2]. This can be explained by the fact that the sample was divided between regular and irregular schedules (without further investigation of shift schedules). In addition, one group was significantly larger than the other.

The works by Jung et al. [17] and Kim et al. [40] have already observed that night work increases the incidence of appetite disorders and impacts physical and psychological well-being. Furthermore, it has also been established [41] that there are different dimensions of eating behaviour that are related to emotional eating, and this is associated with a deficit in impulse control [42], characteristic of uncontrolled eating and the ED subtype BN.

These results highlight that it is necessary to emphasize that preventive measures should be taken to minimize the pathological conditions that may arise as a result of shift work. Therapeutic strategies should revolve around the way in which work is organised and the autonomy in decision-making of each person, in terms of family and social status, as well as health surveillance. In addition, healthy eating behaviour prevention programmes should be implemented. This would mitigate the detrimental effects of low adherence to the MD and EDs.

This study has some limitations. Firstly, it is a cross-sectional study, which does not allow us to establish causal relationships between the study variables. Future research should carry out a longitudinal study in order to look deeper into the subject. Furthermore, it should be noted that the data are self-referenced; thus, there could be a bias in the responses. On the other hand, no men participated in the study, so these data are only applicable to female nurses. It should also be noted that few studies have analysed these variables in nursing staff and, as a consequence, the studies compared do not use the same questionnaires to assess adherence and eating habits. However, it is consistent with current evidence that shift workers’ eating habits suffer from alterations in the quality, quantity and timing of meals. These unhealthy eating habits can lead to an imbalance in the feeling of satiety and, together with negative emotions, may favour the development of EDs.

Rotational shifts may compromise the mental health of nursing staff in relation to eating habits, affecting the quantity and quality of the food intake (macro and micronutrients). For this reason, in future research, the possibility of a prolonged follow-up could be considered to study the changes that the nursing staff present in relation to different variables and health indicators, such as sleep disturbance, changes in body composition, circulating parameters, heart rate variability, etc.

## 5. Conclusions

In conclusion, in this study the adherence to a MD in nursing staff is seen to be average or poor, with no differences depending on the work shift they perform. However, nurses on the RS7 work pattern show a greater avoidance of fattening foods, preoccupation with thinness, symptomatology of bulimia and preoccupation with the foods they eat. This leads to an increasing possibility of manifesting an ED, especially in terms of the establishment of restrictive behaviours, as well as risky eating behaviours or bulimic behaviours, related to an ED.

This information should be taken into account in order to develop programmes for healthy eating habits and the prevention of EDs in nursing staff to improve their quality of life and job performance.

## Figures and Tables

**Table 1:** Description of the nursing staff included in the study.

	Total (*N* = 240)	FMS (*n* = 78)	RS7 (*n* = 82)	RS12 (*n* = 80)
Age (years old)	37 ± 10	37 ± 10	38 ± 11	37 ± 10
Weight (kg)	63.5 ± 10.4	63.5 ± 8.9	64.3 ± 10.0	62.8 ± 12.1
Height (cm)	165 ± 6.4	164.3 ± 6.4	164.5 ± 6.6	165.2 ± 6.1
BMI (kg/m^2^)	23.5 ± 3.6	23.5 ± 3.2	23.9 ± 3.6	23.0 ± 3.8
PREDIMED total score	7.2 ± 2.6	7.8 ± 2.7	7.1 ± 2.3	6.6 ± 2.7

BMI: Body mass index; FMS: Fixed morning shift: RS7: 7 h Rotating shift; RS12: 12 h Rotating shift; *N*: size of the study population; *n*: sample size (group).

**Table 2:** PREDIMED PLUS index statistics for total sample.

	FMS				RS7				RS12			
	No	(%)	Yes	(%)	No	(%)	Yes	(%)	No	(%)	Yes	(%)
Item 1	34	(43.6%)	44	(56.4%)	31	(37.8%)	51	(62.2%)	32	(40.0%)	48	(60.0%)
Item 2	45	(57.7%)	33	(42.3%)	43	(52.4%)	39	(47.6%)	37	(46.3%)	43	(53.8%)
Item 3	53	(67.9%)	25	(32.1%)	53	(64.6%)	29	(35.4%)	58	(72.5%)	22	(27.5%)
Item 4 *	13	(16.7%)	65	(83.3%)	31	(37.8%)	51	(62.2%)	47	(58.8%)	33	(41.3%)
Item 5	35	(44.9%)	43	(55.1%)	42	(51.2%)	40	(48.8%)	43	(53.8%)	37	(46.3%)
Item 6	35	(44.9%)	43	(55.1%)	48	(58.5%)	34	(41.5%)	38	(47.5%)	42	(52.5%)
Item 7	59	(75.6%)	19	(24.4%)	61	(74.4%)	21	(25.6%)	60	(75.0%)	20	(25.0%)
Item 8 *	22	(28.2%)	56	(71.8%)	51	(62.2%)	31	37.8%)	69	(86.3%)	11	(13.8%)
Item 9	66	(84.6%)	12	(15.4%)	70	(85.4%)	12	(14.6%)	73	(91.3%)	7	(8.8%)
Item 10	56	(71.8%)	22	(28.2%)	60	(73.2%)	22	(26.8%)	60	(75.0%)	20	(25.0%)
Item 11	54	(69.2%)	24	(30.8%)	59	(72.0%)	23	(28.0%)	55	(68.8%)	25	(31.3%)
Item 12	72	(92.3%)	6	(7.7%)	79	(96.3%)	3	(3.7%)	75	(93.8%)	5	(6.3%)
Item 13	51	(65.4%)	27	(34.6%)	53	(64.6%)	29	(35.4%)	57	(71.3%)	23	(28.7%)
Item 14	43	(55.1%)	35	(44.9%)	46	(56.1%)	36	(43.9%)	45	(56.3%)	35	(43.8%)
Item 15 *	13	(16.7%)	65	(83.3%)	14	(17.1%)	68	(82.9%)	4	(5.0%)	76	(95.0%)
Item 16	50	(64.1%)	28	(35.9%)	52	(63.4%)	30	(36.6%)	56	(70.0%)	24	(30.0%)
Item 17	18	(23.1%)	60	(76.9%)	19	(23.2%)	63	(76.8%)	22	(27.5%)	58	(72.5%)

FMS: Fixed morning shift: RS7: 7 h Rotating shift; RS12: 12 h Rotating shift; * Item with significant differences between groups; No = did not score the item; Yes = the criterion for 1 score has been met.

**Table 3:** Comparison of the total score and subscales of the EAT-26 questionnaire as a function of work shift using ANCOVA (BMI as a covariate).

	FMS (*n* = 78)	RS7 (*n* = 82)	RS12 (*n* = 80)	F	*p*	η^2^*p*
	X ± SD	X ± SD	X ± SD			
Restriction	2.8 ± 3.6 *	6.1 ± 5.6 *^,#^	3.6 ± 4.9 ^#^	14.54	<0.001	0.07
Bulimia	0.3 ± 0.7 *	1.4 ± 2.1 *^,#^	0.8 ± 1.4 ^#^	0.01	0.022	0.01
Oral control	1.1 ± 1.8	1.7 ± 1.7	1.1 ± 1.8	0.07	0.799	0.00
EAT-26 Total	4.3 ± 4.8 *	9.1 ± 7.9 *^,#^	5.5 ± 7.1 ^#^	6.57	0.011	0.03

BMI: Body mass index; FMS: Fixed morning shift: RS7: 7 h rotating shift; RS12: 12 h rotating shift; η^2^*p*: Partial Eta squared (effect size); X: Mean; SD: Standard deviation; n: sample size (group); *p*: *p* value; F: F statistic; *: significant difference between groups (FMS vs. RS7); ^#^: significant difference between groups (RS7 vs. RS12); both cases with a Bonferroni *p* value < 0.05.

## Data Availability

The data presented in this study are available on request from the corresponding author. The data are not publicly available due to the fact that they are contain personal health information.
